# Results from a Human Factor Study Supporting Safe and Effective Use of the Lecanemab Subcutaneous Autoinjector

**DOI:** 10.1002/alz70859_106273

**Published:** 2025-12-25

**Authors:** Erica Andreozzi, Steven Hersch, Michelle Gee, Michael C. Irizarry, Lynn D Kramer

**Affiliations:** ^1^ Eisai Inc., Nutley, NJ USA; ^2^ Eisai Ltd., Hatfield United Kingdom

## Abstract

**Background:**

Lecanemab is a novel, humanized immunoglobulin G (IgG) 1 monoclonal antibody, which has demonstrated the ability to substantially reduce markers of amyloid and slow clinical decline in early Alzheimer’s disease (AD). Subcutaneous administration using an autoinjector improves convenience and eliminates burden on patients, providers and healthcare systems necessitated by intravenous infusion, thereby broadening access. Herein, we share results from the human factors (HF) program implemented to support the lecanemab autoinjector clinical program.

**Methods:**

The HF validation study evaluated whether the lecanemab autoinjector could be used safely and effectively – with and without training – under expected use environments. It characterized the type, frequency, and severity of potential use errors (including incomplete dosing) as well as labelling comprehension. The study participants (n=110; patients with self‐ or caregiver‐reported diagnosis of mild cognitive impairment or mild AD [n=63], caregivers [n=32], and healthcare providers [HCPs; n=15]) represented lecanemab autoinjector end users and included a mix of injection‐experienced and injection‐naïve. The patients were administered the Mini Mental State Examination (MMSE) and subsequently divided into low MMSE (22‐26) and high MMSE (27‐30) groups. Patients and caregivers were evaluated under trained and untrained conditions, while HCPs were always untrained.

**Results:**

Overall, 94.5% (n=104/110) of participants were successful at delivering the first of 2 injections: untrained, low MMSE patients (93.3%, n=14/15); trained, low MMSE patients (100%, n=15/15); untrained, high MMSE patients (100%, n=16/16); trained, high MMSE patients (94.1%, n=16/17); untrained caregivers (100%, n=15/15); trained caregivers (100%, n=17/17); HCPs (100%, n=15/15). Furthermore, 82.7% (n=91/110) of participants were successful at delivering 2 consecutive injections: untrained, low MMSE patients (53.3%, n=8/15); trained, low MMSE patients (93.3%, n=14/15); untrained, high MMSE patients (93.8%, n=15/16); trained, high MMSE patients (88.2%, n=15/17); untrained caregivers (73.3%, n=11/15); trained caregivers (94.1%, n=16/17); HCPs (80%, n=12/15). Training increased injection success in caregivers and low MMSE patients. Critical information in the labelling was understood by 91.8% (n=101/110) of overall participants. No device malfunctions or harmful events occurred.

**Conclusions:**

The HF validation study confirms that the lecanemab autoinjector is safe and effective for the intended users, uses, and use environments.

